# Rheumatic heart disease knowledge and associated factors among nurses working in cardiac centers at public and private hospitals of Addis Ababa: cross sectional study

**DOI:** 10.1186/s12912-022-00910-5

**Published:** 2022-05-27

**Authors:** Tesfaye Techane, Bethlehem Legesse, Yohannes Ayalew, Aklil Hailu

**Affiliations:** 1 School of Nursing, Department of Medical Surgical Nursing, Saint Paul’s Hospital Millennium Medical College, Addis Ababa, Ethiopia; 2 Addis Ababa University College of Health Sciences School of Nursing and Midwifery, Addis Ababa, Ethiopia

**Keywords:** Knowledge, Rheumatic, Heart, Disease, Nurses

## Abstract

**Background:**

It is proposed that the biggest gap in control of rheumatic heart disease is in implementing of ineffective primary and secondary preventive measures. These measures are supposed to be well addressed by nurses. For prevention and proper management, nurses are expected to have full knowledge about rheumatic heart disease. Therefor the main objective of the study was to assess the level of nurse’s knowledge and factors behind regarding RHD in the current study.

**Method:**

Institution based cross sectional study was conducted on nurses working in cardiac centers of public and private hospitals at Addis Ababa from April 1 to 30, 2021. Total sample size is 163 selected by purposive sampling method. Data was entered in to Epi-data version 4.5 and exported to SPSS version 25.0 and was checked for missing values. Data was cleaned. Descriptive statistics such as frequency, mean and percentages were calculated, described and displayed in tables, graphs and charts. Binary logistic regression was done to see the crude significant relation of each independent variable with nurse’s good knowledge score. Significant factors were identified based on multivariate logistics regression in 95% confidence level at P-value less than 0.05.

**Result:**

In the present study about 154 participants were participated. The mean correct answer response of the nurses for knowledge of RHD questions is 12.2 ± 5.2. Only 48.7% of the nurses have good knowledge towards RHD. Being male in gender, having history of sore throat, taking formal education in university or collage, taking in-service training on RHD, having higher wok experience, have found significantly associated with higher odds of nurses’ good knowledge towards RHD.

**Conclusion and recommendation:**

Regular training regarding RHD management should be given to nurses who are working in cardiac centers. Rheumatic heart disease early treatment and prevention should be incorporated and reinforced in to nursing and other health related professions curriculums.

## Introduction

Rheumatic heart disease (RHD) is a serious disease of the heart involving damage to one or more of the four heart valves [1]. Rheumatic heart disease is a result of rheumatic fever that follows an untreated group A streptococcal infection of young susceptible individuals [2]. The disease is caused by inflammatory and autoimmune reactions [3].

The heart valves injury remains after an illness called acute rheumatic fever, during which the heart valve tissue, also occasionally the heart lining or muscle can become swollen, which leads to carditis [4]. Subsequently, the damaged heart valves become scarred [5]. A scared heart valve leads to an interruption of normal blood flow [6], decreased blood flow, leak in the valve or flow in the wrong direction, and damage to the heart muscle [7], affecting its ability to pump [8]. Damage to the mitral valve or other heart valves and tissues [9] causes an irregular and chaotic heartbeat (Atrial Fibrillation) [10] and Heart Failure (HF) [11].

Rheumatic heart disease is caused by Group A beta hemolytic Streptococci (GAS)which is known for causing acute rheumatic [12]. Rheumatic fever most often affects children between 5 and 15 years old, though it can progress in younger children and adults. Rheumatic fever causes chronic damage to the heart (Rheumatic Heart Disease) [13]. It generally occurs 10 to 20 years after the first illness, but severe cases of rheumatic fever can cause damage to the heart valves without showing symptoms [14].

Numerous factors increase the risk of rheumatic fever [15]. Family history and strain of streptococcus bacteria are the most commonly stated factors [16, 17]. However, the greater risk of rheumatic fever is associated with overcrowding, poor sanitation, and other conditions that can easily result in the rapid transmission or multiple exposures to streptococcus bacteria [18, 19].

Rheumatic heart disease is a preventable but a major public health problem in low- and middle as well as high-income countries [18]. More than 30 million people are affected by rheumatic heart disease nowadays worldwide [19]. According to WHO 2015 report, RHD caused 305,000 deaths and 11.5 million disability-adjusted life years lost [20]. From these deaths, 60% happened before the age of 70 years [21].

The African, South-East Asia, and the Western Pacific regions are extremely affected, accounting for 84% of all prevalent cases and 80% of all estimated deaths due to rheumatic heart disease in 2015 [22]. India, in the South-East Asia region, has the highest global prevalence, with about 27% of all cases globally [23]. In the Western Pacific Region, the burden of rheumatic heart disease is especially concentrated in China and indigenous populations living in Australia, New Zealand, and the Pacific Island States [23]. In the Eastern Mediterranean Region, rheumatic heart disease persists in certain countries such as Egypt, Sudan, and Yemen [24].

Rheumatic heart disease is prevalent in our country Ethiopia, where an average of 40% of cases per 1000 population is affected [25]. Around 62% of males and 53% of the female population experienced RHD in south west Ethiopia [26].

The possible barriers to prevention, control, and elimination of rheumatic heart disease are the neglect of rheumatic fever and rheumatic heart disease in national health policies and budgets [27], lack of data to enable pointing prevention efforts [28], poor primary and secondary prevention, and access to primary health care [29], inadequate numbers, training or knowledge of health workers at all levels [30], limited understanding of rheumatic fever and or rheumatic heart disease in affected communities and inaction on the social determinants of the disease and inequities in health [31].

It is proposed that the biggest gap in control of rheumatic heart disease is ineffective primary and secondary preventive measures. These measures are supposed to be well addressed by health care providers especially nurses [32]. For prevention and proper management of RHD health care providers are expected to have a full understanding of the nature of the disease condition and good assessment knowledge [33]. Thus, for effective prevention and management of RHD nurses must be well-educated and knowledgeable about the diseases [34].

Although RHD can be prevented and effectively managed, the prevalence of the disease is still increasing. Knowledgeable nurses are very crucial in reducing the prevalence of RHD by providing health education and quality care. In Ethiopia little is known about the level of knowledge of nurses towards RHD. Therefore, the main objective of the study was to assess nurses’ knowledge about Rheumatic heart disease in Addis Ababa public and private hospitals.

## Methods and materials

### Study setting

This study was conducted in Addis Ababa public and private hospitals with cardiac units. According to the data obtained from Addis Ababa City Administration Health Bureau, there are 6 public and private cardiac hospitals in Addis Ababa, which were giving cardiac care services. All were purposely selected to be the study areas; these are Tikur Anbessa Specialized Hospital (TASH) cardiac center, Saint Peter hospital (SPH), Cardiac Center Ethiopia (CCE), Land Mark Hospital (LMH), Gesund hospital (GH), and Addis Cardiac Hospital (ACH). These hospitals provide cardiac care services to patients with diverse socio-economic background. Besides, there is a high load of rheumatic heart disease patients in these hospitals and the only places where cardiac nurses are working in the city.

### Study design

A health facility-based cross-sectional study with a quantitative research method was employed to address the specific objectives of the study from April 1 to 30 2021.

### Inclusion and exclusion criteria

- Inclusion criteria: Nurses who are working in Addis Ababa public and private hospitals with cardiac units and willing to participate in the study were included.
- Exclusion criteria: Nurses who were seriously sick and unavailable during the data collection period.

### Sample size determination and sampling procedures

**Fig. 1:**
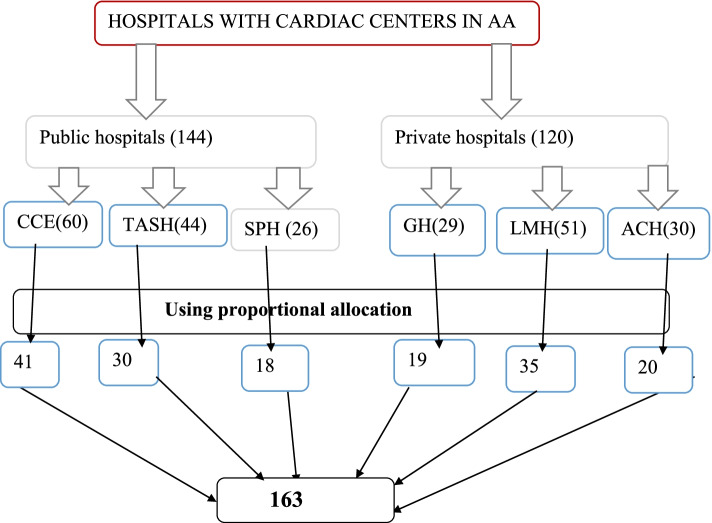
Schematic presentation of sampling technique in the selected public and private hospitals with cardiac center in Addis Ababa Ethiopia 2021 (the numbers in the brackets represents, the total number of cardiac nurses in respective hospitals, or group of hospitals (public or private) and the numbers in the boxes represents the total number of nurses included in the study from respective hospitals after proportional allocation is made. And 163 shows the total number of potential study participants). Key;—AA: Addis Ababa, CCE: Cardiac Center Ethiopia, TASH: Tikur Anbessa Specialized Hospital, SPH: St. Peter Hospital, GH: Gesund Hospital, LMH: Land Mark Hospital, ACH: Addis Cardiac Hospital

The sample size was determined by using the formula for estimating a single population proportion formula. Since the population size is less than 10, 000, the final sample size was estimated using the correction formula. The final sample size obtained including a 10% non-response rate was (163). Then, the number of participants in each selected hospital was determined using the population proportionate sampling (PPS). It is estimated using the formula: (T) = K*L, (K*HL in a health facility)/K_,_ where, HL = proportion of cardiac nurses in the study in a given hospital, T = Final sample size obtained using correction formula (163), K is the total number of cardiac nurses in the selected hospitals. Finally, nurses were selected using a convenience sampling method [Fig. 1].

### Study instrument

Data was collected using a self-administered questionnaire for nurses’ knowledge about RHD. The tool has two parts.

1. Sociodemographic variables include (Age, Sex, Religion, Work experience, Educational level, Salary, etc.…)
2. RHD knowledge of nurses questionnaire (was first adopted from a study conducted in Cameroon [32] and adapted for this study. Nurse experts and a senior physician reviewed and approved the questionnaire before actual data collection. The RHD tool contains 24 questions, while scoring was done giving “1” point for each correct answer with the maximum score of 24).

Pilot testing was done in Saint Paul’s Hospital Millennium Medical College (SPHMMC) among 10 nurses. Necessary revisions were made after pilot testing.

### Operational definition

In the current study, the mean correct response of the participants was 12.2

**Good knowledge**: if participants score mean and above (≥ 12.2) to RHD knowledge questions.

**Poor knowledge**: if the participants score below mean (< 12.2) to RHD knowledge questions.

**Cardiac center nurses**: in this study, nurses who are working in cardiac centers including those who are accredited as cardiac nurses were included in this study.

### Data analysis

Data were entered into Epi-data version 4. 45 software and exported to SPSS version 25.0 for analysis and checked for missing values. Data were cleaned. Normality was checked using frequency histogram and kolmogorove-smirnov test was employed. In the kolmogorove-smirnov (K-S) test the p value for all the variables were above 0.05 and the skewness and kurtosis below 1 (near to zero). The data were found normally distributed. Descriptive statistics, such as mean and standard deviation was used to compute continuous variables and frequency and percentage for categorical variables. Both bivariate and multivariate logistic regression analyses were fitted to identify independently associated factors with nurses’ knowledge about RHD. Multivariate analysis was employed at P ≤ 0.05 to control the possible effect of confounding. Adjusted odds ratio (AOR) with 95% CI were used to select variables associated with nurses’ knowledge about RHD.

## Results

### Socio-demographic characteristics of the participants

In the present study, 154 participants were participated, with a response rate of 154(95%). The mean age of the participants was 28.8 years ranging from 21 to 44. More than three-fourths 128 (83.1%) of the participants were females, while 92(59.7%) of the nurses were in the age group of 21 – 28 years old category.

One hundred five (68.2%) of the study participants were BSc degree holders whereas only 17(11%) had a Master’s degree in the nursing field. Regarding marital status, 80(51.9%) were single and 66(42.9%) of them were married as few as 8(5.2%) of the study participants were widows or divorced. The majority of the participants were from Land Mark Hospital 8(24.7%) and St. Peter hospital participants 15(9.7%) cover the smallest number.

The nurses working in the selected study area’s mean income was 6248.07 ± 2391.5 Ethiopian Birr and the majority of the study participants 105(68.2%) earn less than the mean income (6248.07 ETH Birr). Most 72(46.8%) of them had work experience from two to five years and 52(33.8%) had a sore throat at least once in their life. Twenty-eight (18.2%) of the nurses had been diagnosed with rheumatic heart disease 92(59.2%) have experience in managing RHD patients. Nearly 80% (123) and 35(22.7%) of the nurses working in the current study area had learned formal education in university about RHD and took in-service training on RHD after start working respectively (Table 1).

**Table 1:** Socio demographic characteristics of nurses working in public and private cardiac center hospitals at Addis Ababa, Ethiopia 2021

Variables	Category	Frequency(n)	Percentage (%)
Sex	Male	26	16.9
	Female	128	83.1
Age	21- 28 years	92	59.7
	29-36 years	44	28.6
	> 37 years	18	11.7
Level of educational	Diploma	32	20.8
	BSc nurse	105	68.2
	MSc nurse	17	11
Religion	Orthodox Christian	90	58.4
	Muslim	34	22.1
	Protestant	27	17.5
	Catholic	3	1.9
Marital status	Single	80	51.9
	Married	66	42.9
	Divorced	4	2.6
	Widowed	4	2.6
Current working place (hospital)	TASH	28	18.2
	St. Peter Hospital	15	9.7
	Cardiac Center Ethiopia	33	21.4
	Gezund Hospital	20	13
	Land Mark Hospital	38	24.7
	Addis Cardiac hospital	20	13
Year of experience in care provision (years)	< 1 years	31	20.1
	years	72	46.8
	> 6 years	38	24.7
Have you ever had a sore throat?	Yes	52	33.8
	No	102	66.2
Have you been diagnosed with rheumatic heart disease?	Yes	28	18.2
	No	126	81.8
Do you have experience in managing RHD patients?	Yes	92	59.2
	No	62	40.3
Have you learned formal education in your university about RHD?	Yes	123	79.9
	No	31	20.1
Have you taken in service trainings on RHD after you start working?	Yes	35	22.7
	No	119	77.3
Monthly income	< 4500 Eth Birr	37	24
	4501–7500 Eth Birr	76	49.4
	> 7501 Eth Birr	41	26.6

### Nurses’ knowledge towards Rheumatic heart disease (RHD)

As shown in (Table 2), more than half 84(54.5%), of the nurses answered correctly for the question item “What causes rheumatic heart disease?” and 114(74%), answered correctly for the question “can a sore throat cause heart disease?”.

**Table 2:** knowledge towards Rheumatic heart disease (RHD) among nurses working in public and private cardiac center hospitals Addis Ababa Ethiopia 2021

R.n	Knowledge of RHD items	Correct answers		Wrong answers	
		Frequency (n)	Percent (%)	Frequency (n)	Percent (%)
1	What causes rheumatic heart disease?	84	54.5	70	45.5
2	What is the clinical manifestation of acute rheumatic fever?	70	45.5	84	54.5
3	Can a sore throat cause heart disease?	114	74	40	26
4	Which strain of the germ is implicated?	56	36.4	98	63.3
5	What is the duration from a sore throat to the onset of acute rheumatic fever?	46	29.9	108	70.1
6	Within which time range does the treatment of sore throat have to be initiated to reduce the risk of acute rheumatic fever?	36	23.4	118	76.6
7	Which treatment is appropriate for a bacterial sore throat for prevention of acute rheumatic fever and rheumatic heart disease?	131	85.1	23	14.9
8	Carditis in acute rheumatic fever most often persists with the resolution of other symptoms	107	69.5	47	30.5
9	Rheumatic heart disease can occur without prior evidence of acute rheumatic fever	80	51.9	74	48.1
10	Which lesion is commonly associated with carditis in acute rheumatic fever?	40	26	114	74
11	Can indolent carditis alone fit the criteria for the diagnosis of acute rheumatic fever?	50	32.5	104	67.5
12	Patients with acute rheumatic fever or rheumatic heart disease should be put on secondary prophylactic antibiotics	105	68.2	49	31.8
13	What is the drug of choice for secondary prophylaxis?	87	56.5	67	43.5
14	What is the frequency of prophylaxis with Benzathine Penicillin?	96	62.3	58	37.7
15	What is the minimum duration of prophylaxis?	32	20.8	122	79.2
16	What are the complications of rheumatic heart disease?	73	47.4	81	52.6
17	Carditis in acute rheumatic fever is treated with?	41	26.6	113	73.4
18	Which valve is most commonly involved in rheumatic heart disease?	113	73.4	41	26.6
19	which pone is the earliest valve lesion?	57	37	97	63
20	Should some patients with rheumatic heart disease be placed on anticoagulants?	100	64.9	54	35.1
21	Early treatment of bacterial pharyngitis with antibiotics	123	79.9	31	20.1
22	What the problems with benzathine penicillin injections	107	69.6	47	30.5
23	Which of the following is characteristics of bacterial tonsillitis	44	28.6	110	71.4
24	Early treatment of bacterial pharyngitis with antibiotics	86	55.8	68	44.2

The nurses were asked about the appropriate treatment of choice for a bacterial sore throat and prevention of acute rheumatic fever or rheumatic heart disease, and 131(85.1%) of them provide correct answer to the question. Significant number 107(69.5%), of the nurses provide correct answer to the question “Carditis in acute rheumatic fever most often persists with the resolution of other symptoms” and more than half 80(51.9%) of the study participants answered correctly to the question stating about rheumatic heart disease occurrences without prior evidence of acute rheumatic fever.

Similarly, 105 (68.2%) of the participants answered correctly to the question “patients with acute rheumatic fever or rheumatic heart disease should be put on secondary prophylactic antibiotics”. Only 87(56.5%) of the participants knew “what the drug of choice for secondary prophylaxis is”. In this study, 96(62.3%), 100(64.9%), and 113(73.4%) of the study participants answered correctly to the questions that enquired “the frequency of prophylaxis with Benzathine Penicillin, “the valve that most commonly involved in rheumatic heart disease” and if “patients with rheumatic heart disease should be placed on anticoagulants” respectively.

As much as 123(79.9%), of the participants provide correct response to the question if “early treatment of bacterial pharyngitis with antibiotics is possible” and 107(69.6%) of the study participants knew the problems with benzathine penicillin injections. Early treatment of bacterial pharyngitis with antibiotics 86(53.8%)?” correctly.

Only 32(20.8%) of the study participants correctly knew “what the minimum duration of prophylaxis is”.

Based on this the mean correct answer score of the nurses for knowledge of RHD questions is 12.2 ± 5.2, with a minimum 0 and maximum score of 23 out of 24 question items.

**Fig. 2:**
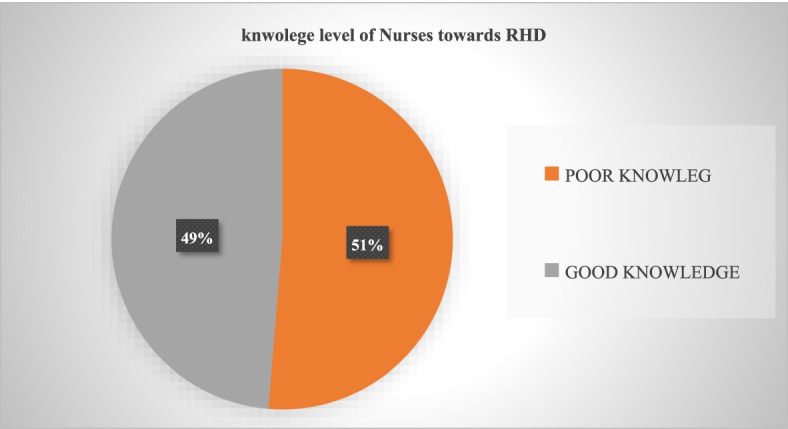
A pie chart showing nurses knowledge towards RHD at public and private hospitals with cardiac centers in Addis Ababa Ethiopia 2021

And therefore based on the current study 75(48.7%) of the nurses have scored above mean to the knowledge of RHD questions and 79(51.3%) have scored below the mean to nurses’ knowledge questions. Only 48.7% of the nurses have good knowledge of RHD among nurses who are working in public and private hospitals with the cardiac centers in Addis Ababa, Ethiopia ([Fig. 2)].

### Nurses’ knowledge towards RHD and associated factors

To identify factors associated with nurses’ good knowledge towards RHD, a logistic regression model was fitted. On bivariate logistic regression analysis, being male in gender, having a history of sore throat any time before, taking formal education in university or college about RHD, taking in-service training on RHD, being diagnosed for RHD, having experience of managing RHD patients, has found an association with higher odds of good knowledge towards RHD in the current study. Finally, after obtaining statistically significant variables at *p* < 0.05 in bivariate logistic regression analysis, a multivariate logistic regression analysis was carried out to see the independent predictors of good knowledge towards RHD. The multivariate logistic regression was carried out by taking good knowledge towards RHD as a covariate in addition to those variables where the significant association was obtained in bivariate logistic regression. After adjusting potential cofounders, being male in gender, having a history of sore throat any time in life, taking formal education in university or college about RHD, taking in-service training on RHD, having higher work experience, have been found significantly associated with higher odds of nurses’ good knowledge towards RHD at p values less than 0.05 (Table 3).

**Table 3:** knowledge towards RHD and its associated factors among nurses working in public and private cardiac centers in Addis Ababa, Ethiopia 2021

Characteristics	Category	Knowledge towards RHD		*P*-value	COR (Lower and upper limit)	*P*-value	AOR (Lower and upper limit)
		Poor n (%)	Good n (%)				
Sex	Male	7(4.5%)	19(12.3%)	0.009^*^	3.5(1.37,8.89)	0.017*	4.6(1.33,16.04)
	Female	72(46.8%)	56(36.4) %		1		1
Age	21–28 years	55(35.7%)	37(24%)	0.045*	.34(.116,.976)	0.995	1(0.16,6.23)
	29–36 years	18(11.7%)	26(16.9%)	0.579	.72(.23,2.28)	0.861	1.16(.21,6.39)
	> 37 years	6(3.9%)	12(7.8%)		1		1
Work experience	< 1 years	26(18.4%)	5(3.5%)	0.000*	.089(.027,.28)	0.001*	.057(.011,0.3)
	years	31(22%)	41(29.1%)	0.23	.61(.26,1.397)	0.306	0.54(0.17,1.75)
	> 6 years	12(8.5%)	26(18.4%)		1		1
Monthly income	< 4500 ETB	25(16.2%)	12(7.8%)	0.001*	.2(.076,0.52)	0.552	0.63(.137,2.89)
	4501–7500	42(27.3%)	34(22.1%)	0.008*	.34(.15,.753)	0.251	.46(.125,1.722)
	> 7501 ETB	12(7.8%)	29(18.8%)		1		1
sore throat	Yes	15(9.7%)	37(24%)	0.00*	4.1(2.01,8.55)	0.001*	5.8(2.04,16.53)
	No	64(41.6%)	38(24.7%)		1		1
Diagnosed with rheumatic heart disease	Yes	8(5.2%)	20(13%)	0.01*	3.2(1.3,7.87)	0.49	1.6(0.42,6.24)
	No	71(46.1%)	55(35.7%)		1		1
Experience in managing RHD patients	Yes	39(25.3%)	53(34.4%)	0.008*	2.47(1.27,4.8)	0.22	1.8(0.69,4.82)
	No	40(26%)	22(14.3%)		1		1
Learned formal education in your university about RHD?	Yes	57(37%)	66(42.9%)	0.017*	2.8(1.27,6.64)	0.039*	4.3(1.07,17.5)
	No	37(14.3%)	9(5.6%)		1		1
Training on RHD	Yes	7(4.5%)	28(18.2%)	0.000*	6.1(2.47,15.1)	000*	10.9(2.93,40.6)
	No	72(46.8%)	47(30.8%)		1		1
	> 7501 ETB	12(7.8%)	29(18.8%)		1		1

1: constant, *Significant at *p*-value of < 0.05

## Discussion

The present study assesses the knowledge level of nurses towards Rheumatic Heart Disease (RHD) and associated factors in Addis Ababa public and private hospitals with cardiac units. Subsequently, the mean correct answer response of the nurses for knowledge of RHD questions is 12.2 ± 5.2, with a minimum 0 and maximum score of 23 out of 24 question items. This is consistent compared to a study conducted in four medical schools in Cameroon [32] where the mean knowledge score was 11.97 out of 24 questions.

In the current study 75(48.7%) study subjects had good knowledge about RHD. This is higher compared to a hospital-based cross-sectional study conducted in, (38%) Sudan [31]. This discrepancy may be due to better work experience in Cardiac clinics in the current study or exposure to rheumatic disease imposes a better perception towards RHD.

The current study finds out that the more than half 79(51.3%) of the study subjects have poor knowledge of RHD. This is consistent compared to a cross-sectional study conducted in Gezira State, Sudan [35]. The study found that majority of the health worker’s knowledge towards RHD were found to be poor.

Our study finds out that 87(56.5%) and 96(62. 3%) of the participants know “What the drug of choice for secondary prophylaxis is “, and the frequency of prophylaxis with Benzathine Penicillin respectively.

This agrees with a study conducted in Cameroon [32]. The drug of choice for secondary prophylaxis was known by 74.5% of study subjects, and 84.7% responded that Benzathine penicillin is the drug of choice for the treatment of sore throat to prevent acute rheumatic fever.

In the current study about 114(74%), know that amoxicillin is appropriate for a bacterial sore throat for the prevention of acute rheumatic fever and rheumatic heart disease. However, a study conducted in Cameroon [32] revealed that 30.1% of study participants thought that amoxicillin was the drug of choice for secondary prophylaxis for acute rheumatic fever or rheumatic heart disease. This discrepancy may be due to differences in socioeconomic status and or lack of in-service training about RHD in the later study.

The current study identified that taking formal education in university or collage about RHD, taking in-service training on RHD, having higher wok experience, have found significantly associated with higher odds of nurses’ good knowledge towards RHD. This partially agrees with the world heart federation second edition report [36], which explored that “health worker training has a central role in RHD control programs” and “provision of training whether in the job or scheduled to health care providers on rheumatic heart diseases have shown to increase the knowledge level of the professionals on rheumatic heart disease.” The study has not mentioned whether or not having higher work experience could improve nurses’ knowledge level.

In this study male nurses working in the cardiac unit of the current study area were having 4.6 times higher odds of good knowledge towards RHD compared to female nurses ([AOR = 4.6, 95% CI (1.33–16.045)) *P* = 0.017)]. Most kinds of literature [34–37], didn’t support this idea. This may be due to the fact that difference in socio-cultural background of the nurses in the studies.

In this study nurses’ who had formal education towards RHD in college or university have 4.3 times more likely higher odds of better knowledge towards RHD ([AOR = 4.3, 95% CI (1.07,17.5)) *P* = 0.039)]. This finding agrees with studies conducted in Sudan, Gaafar Ibnauf Children’s Hospital [31] and another cross-sectional study conducted in Cameroon [32] which explored having a formal lecture on RHD contributes to higher nurses’ RHD knowledge.

The current study identified that taking formal education in university or collage about RHD, taking in-service training on RHD, having higher wok experience, have found significantly associated with higher odds of nurses’ good knowledge towards RHD. In addition, study subjects who had a history of a sore throat had shigher knowledge level compared to those who have never had sore throat previously. This is consistent compared to studies conducted in Fuji [37], Sudan [35] and Cameron [32] which explored that health workers’ knowledge towards ARF/RHD were significantly associated with taking training on RHD, public education about RHD except nothing was mentioned about the history of sore throat and RHD knowledge relation. This may be due to the fact that nurses in the later studies may have been taken the prophylactic treatment before exposed to RHD.

Study participants with short work experience period/ time/ have associations with a low level of nurses’ good knowledge of RHD. Participants with less work experience had 0.57 times less probability of having better knowledge towards RHD compared to those who had a long period of work experience [AOR = 0.57: 95% CI (0.57(0.011,0.3)) *P* = 0.001)]. This may be due to nurses extensive practice repertoires, doing the same job repetitively for prolonged time at cardiac centers leads to acquire more knowledge about RHD.

## Conclusion and recommendations

The mean correct answer response of the nurses for knowledge of RHD questions is 12.2 ± 5.2, with a minimum 0 and maximum score of 23. In this study, 75(48.7%) of the nurses have scored above mean to the knowledge of RHD questions and 79(51.3%) have scored below mean to nurses’ knowledge questions. Only 48.7% of the nurses have good knowledge of RHD among the nurses who are working in public and private hospitals with the cardiac centers in Addis Ababa, Ethiopia.

Being male in gender, having a history of sore throat any time in life, taking formal education in university or collage about RHD, taking in-service training about RHD, having higher wok experience, have found significantly associated with higher odds of nurses’ good knowledge towards RHD. There is a need to ensure that education and in-service training regarding RHD management should be given to nurses who are working in cardiac centers.

### Study limitations

The cross-sectional nature of the study including, limited sample size and non-probability sampling technique make our study difficult to show the Couse effect relationship and generalize or infer the finding. And therefore, we recommend avoiding inferring the finding of the current study outside the mentioned study areas.

## Data Availability

The data analyzed during the current study is available from the corresponding author on reasonable request.

## Abbreviations

ACH: Addis Cardiac Hospital
AF: Atrial Fibrillation
ARF: Acute Rheumatic Fever
CC: Cardiac Center
CCE: Cardiac Center Ethiopia
CI: Confidence Interval
GH: Gesund Hospital
GICH: Gaafar Ibnauf Children Hospital
HF: Heart Failure
KAP: Knowledge, Attitude and Practice
LMH: Land Mark Hospital
RHD: Rheumatic heart disease
SPH: St. Peter Hospital
TASH: Tikur Anbessa Specialized Hospital
USA: United States of America
WHO: World Health Organization
